# Overactive Bladder Symptoms as a Predictor of Longitudinal Decline in Grip Strength in Community‐Dwelling Men: A 4‐Year Longitudinal Study

**DOI:** 10.1111/luts.70056

**Published:** 2026-03-08

**Authors:** Hiroyuki Sato, Teppei Okamoto, Tomoko Hamaya, Hirotake Kodama, Takuma Narita, Jotaro Mikami, Naoki Fujita, Hayato Yamamoto, Atsushi Imai, Koichi Murashita, Shigeyuki Nakaji, Shingo Hatakeyama

**Affiliations:** ^1^ Department of Urology Hirosaki University Graduate School of Medicine Hirosaki Japan; ^2^ Research Institute of Health Innovation Hirosaki University Graduate School of Medicine Hirosaki Japan

**Keywords:** grip strength, male lower urinary tract symptoms, overactive bladder, urinary urgency

## Abstract

**Objectives:**

Lower urinary tract symptoms (LUTS) are associated with sarcopenia in men. However, it is not clear whether LUTS are related to the decline in physical function. We investigated the longitudinal association between the LUTS severity and the grip strength change in community‐dwelling men.

**Methods:**

A 4‐year longitudinal study was conducted using data from 151 men (median age 53 years) enrolled in the Iwaki Health Promotion Project. Grip strength change was calculated as the difference between 2015 and 2019 measurements. LUTS severity was assessed with the International Prostate Symptom Score (IPSS) and the Overactive Bladder Symptom Score (OABSS). Baseline variables included age, Aging Male Symptoms score, serum testosterone, albumin, interleukin‐6, HbA1c, daily exercise, skeletal muscle mass index, and percent body fat. Correlations and multiple regression analyses were performed.

**Results:**

The median age and baseline grip strength were 53 years and 41.3 kg, respectively. Grip strength decreased significantly after 4 years, with a median value of −1.1 kg. There was a significant negative correlation between total OABSS, OABSS Q3 (frequency of urinary urgency, *r* = −0.234, *p* = 0.003), Q4 (frequency of urge urinary incontinence, *r* = −0.190, *p* = 0.020), and grip strength change, whereas the IPSS voiding symptoms domain was not. Multiple regression analysis showed that only the total score of OABSS Q3 and Q4 was significantly correlated with grip strength change (standardized *β* = −0.930, *p* = 0.012).

**Conclusions:**

The main symptoms of OAB may be indicators of the grip strength decline in men.

## 1 Introduction

Lower urinary tract symptoms (LUTS) are symptoms related to problems with the lower urinary tract, including the bladder, prostate, and urethra [1]. Common symptoms of LUTS include hesitancy, weak or intermittent stream, straining, urgency, frequency, nocturia, and incontinence. LUTS can have a negative impact on quality of life, such as sleep disturbance, depression, and decline in physical function [1, 2, 3, 4, 5]. Decline in physical function such as worsening grip strength consequently leads to sarcopenia, which is associated with several adverse health outcomes [6]. Previous studies have suggested a possible association between LUTS and sarcopenia [1, 7, 8, 9]. In particular, physical functions such as walking speed and grip strength are known to be predictors of worsening LUTS [8, 10, 11]. However, few studies have demonstrated that LUTS severity can predict worsening muscle strength. Herein, we longitudinally assessed the relationship between LUTS severity and changes in muscle strength, as measured by grip strength, in community‐dwelling men.

## 2 Materials and Methods

### 2.1 Design and Ethics Statement

The Iwaki Health Promotion Project is an annual health survey initiative that has been conducted since 2005 for the residents in the Iwaki area of Hirosaki city [12]. The project aims to prevent lifestyle‐related diseases, promote a health‐conscious lifestyle, and improve life expectancy among Hirosaki's population. The project has been supported financially by the Japanese government since it was selected as the Center of Innovation program in 2013. The project adheres to the ethical principles outlined in the Declaration of Helsinki, and the utilization of data from the project was granted approval by the Ethics Committee of Hirosaki University School of Medicine (authorization number: 2014‐377‐1 and 2020‐046‐4). This analysis was also approved by the institutional ethical committee (authorization number: 2024‐093‐1). The project is an example of industry‐academia‐government‐private sector collaboration to address the challenges of a super‐aging society. The selection of a 4‐year follow‐up period is consistent with several other population‐based longitudinal studies of grip strength. Previous research has demonstrated meaningful age‐related changes in handgrip over similar intervals [13, 14], supporting the validity of this timeframe as sufficient to capture muscle strength decline in general populations.

### 2.2 Data Collection and Evaluation of Variables

Participants willingly gave their written informed consent and responded to a series of inquiries related to their lifestyle and personal details. Additionally, they underwent various tests, including a blood test. The blood samples were collected in the morning and promptly centrifuged to extract the serum, which was then stored in tubes containing EDTA at −80°C until further analysis. The serum albumin, total testosterone levels, and hemoglobin A1c (HbA1c) were analyzed using conventional laboratory methods. We also obtained serum interleukin‐6 (IL‐6) levels, which are associated with sarcopenia [15]. Estimated glomerular filtration rate (eGFR), hypertension status, use of LUTS‐related medications (α1‐blockers, anticholinergic agents, and β3‐adrenergic agonists), diabetes mellitus (defined by physician diagnosis or use of antidiabetic medications), and history of cardiovascular diseases (stroke, arrhythmia, ischemic heart disease, and valvular heart disease).

In 2015, a total of 431 men participated in the study, out of which 267 continued their involvement in 2019. All baseline data were extracted from the 2015 database. We assigned metabolic equivalents (METs) to the weekly total of MET hours, determined based on the participants' exercise duration [16]. We used the Aging Males' Symptoms (AMS) questionnaire to assess symptoms in aging men [17]. Muscle mass and percent body fat mass were measured using a bioelectrical impedance device. This device provides a non‐invasive and reliable assessment of body composition. The Skeletal Muscle Mass Index (SMI) is a measure of muscle mass calculated by dividing the total muscle mass of the extremities by the square of the height (m). This index is used to assess muscle mass. The study excluded data from individuals who were missing critical information needed for the analyses.

### 2.3 Grip Strength Evaluation

Grip strength is widely recognized as a reliable and practical indicator of overall muscle strength [18]. Muscle strength declines earlier and more rapidly than muscle mass with aging. Therefore, in this study, grip strength was used as a representative and quantitative indicator of change in overall muscle strength. Grip strength was measured using a digital pinch gauge. Participants were instructed to stand in an upright position with their wrist in a neutral position. They were also instructed to relax their hand and maintain a steady position throughout the test. Participants were asked to grip the dynamometer handle as tightly as possible without any other movement or compensation. Each participant performed this test for both hands. The representative value for each year was the mean grip strength of each hand. We calculated the grip strength change by subtracting grip strength in 2019 from grip strength in 2015.

### 2.4 Evaluation of LUTS of Participants

The severity of LUTS was evaluated using the International Prostate Symptom Score (IPSS) and the Overactive Bladder Symptom Score (OABSS). The IPSS is a validated scoring system used to assess the severity of LUTS in patients with benign prostatic hypertrophy (BPH). The IPSS consists of seven questions related to urinary voiding and storage symptoms [19]. The IPSS consists of seven questions (Q1–Q7) related to urinary voiding (Q1, Q3, Q5, and Q6) and storage symptoms (Q2, Q4, and Q7) [19]. Scores range from 0 to 35, with higher scores indicating more severe symptoms. We used the total IPSS voiding symptom domain (Q1, Q3, Q5, and Q6) to assess urinary voiding symptoms. The OABSS consists of four individual questions related to OAB symptoms, each measured on its own scale. Q1 assessed daytime frequency, Q2 evaluated nocturia, Q3 examined urinary urgency (UU), and Q4 addressed urge incontinence (UUI) [20]. The scores from these questions were aggregated and converted to a scale ranging from 0 to 15, where higher scores indicated a more severe OAB condition. Based on the clinical definition of OAB, urinary urgency and urge urinary incontinence (OABSS Q3 and Q4) were considered core symptoms; therefore, the combined Q3 + Q4 score was predefined as the primary explanatory variable.

### 2.5 Statistical Analysis

The statistical analysis was conducted using GraphPad Prism 5.03 (GraphPad Software, San Diego, CA, USA) and EzR: R commander (version 1.6–3). For normally distributed continuous variables, the data were presented as means (standard deviation [SD]), while non‐normally distributed variables were expressed as medians (interquartile ranges [IQR]). Correlation was analyzed with Pearson's rank correlation coefficient. To investigate the factors contributing to the grip strength change, multiple linear regression analysis was conducted. To assess the relative importance of the independent variables and to facilitate comparison among them, standardized *β* coefficients were calculated for each predictor in the multiple linear regression model. Standardized *β* coefficients allow a direct comparison of the effect sizes of different independent variables within the same regression model because they are on a common scale. This facilitates the identification of the most influential predictors in explaining the variability of the dependent variable. The covariates selected for inclusion in the multiple linear regression were determined to account for factors that could potentially influence decline in physical function. These variables included age, aging male symptoms, nutritional status (serum albumin levels), amount of daily exercise, indicators of decline in physical function, serum testosterone and IL‐6 levels, HbA1c, and IPSS [15, 21, 22, 23, 24, 25, 26].

## 3 Results

### 3.1 Baseline Characteristics

**FIGURE 1:**
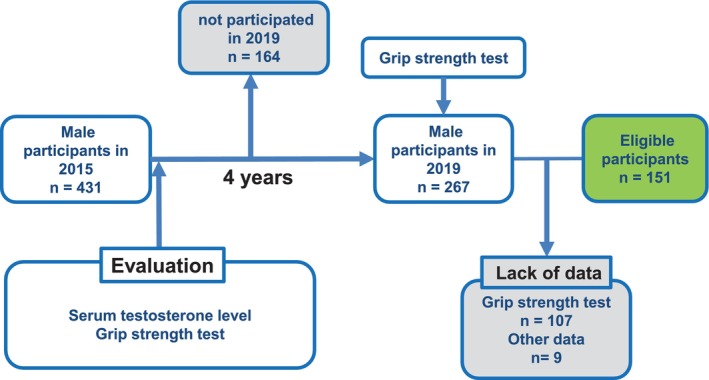
Analyzed participants in this study. Of 431 male participants in the year of 2015, 267 participated in 2019. Of those participants, we excluded 116 who lacked crucial data for further analysis. Finally, we evaluated 151 men.

Of the 431 participants in 2015, 267 participated in 2019. Of these participants, we excluded 116 who lacked crucial data for further analysis. Finally, we evaluated 151 men (Figure 1). Table 1 shows the characteristics of the study participants. The median values of age, baseline total IPSS, OABSS, and grip strength were 53 years, 2 points, 1 point, and 41.3 kg, respectively. At baseline in 2015, 27 of the 151 participants (17.9%) had moderate‐to‐severe LUTS, defined as an IPSS score ≥ 9, and 20 participants (13.2%) met the diagnostic criteria for OAB based on the OABSS. At the 2019 follow‐up, 30 participants (19.9%) had an IPSS score ≥ 9, and 18 participants (11.9%) fulfilled the diagnostic criteria for OAB. Grip strength decreased significantly over the 4 years, with a median decrease of −1.1 kg. Although excluded participants (*n* = 115) tended to be older, baseline LUTS severity and other key clinical variables were generally comparable between the two groups (Table S1).

**TABLE 1:** Baseline characteristics of the study participants.

Number of participants	151
Age (years)	53 (37–63)
BMI (kg/m^2^)	23.2 (21.4–25.1)
SMI (kg/m^2^)	8.1 (7.6–8.8)
Serum albumin level (g/dL)	4.6 (4.4–4.8)
AMS scale	26 (21–32)
eGFR (mL/min/1.73 m^2^)	80.5 (71.4–88.1)
The use of anti‐hypertensive medication (*n*, %)	52 (34%)
LUTS treatment (*n*, %)	4 (2.6%)
History of cardiovascular disease (*n*, %)	8 (5.3%)
Diabetes mellitus (*n*, %)	15 (10%)
HbA1c (%)	5.6 (5.4–5.9)
Total IPSS	2 (1–6)
Total OABSS	1 (0–2)
METs Daily exercise/week	0 (0–3.5)
Serum testosterone level (ng/dL)	589 (446–729)
Percent body fat (%)	19.3 (15.5–22.1)
Serum IL‐6 level (pg/mL)	0.99 (0.68–1.46)
Grip strength in 2015 (kg)	41.3 (36.3–45.5)
Grip strength in 2019 (kg)	39.0 (34.0–44.0)
Grip strength change (kg)	−1.1 (−3.2–0.96)

*Note:* Values are presented as median (Interquartile range).

Abbreviations: AMS, aging males' symptoms; BMI, body mass index; eGFR, estimated glomerular filtration rate; HbA1c, hemoglobin A1c; IL‐6, interleukin‐6; IPSS, International Prostate Symptom Score; LUTS, lower urinary tract symptoms; METs, metabolic equivalents; OAB, overactive bladder; SMI, skeletal muscle mass.

### 3.2 Correlation Between the Grip Strength Change and LUTS Severity

**FIGURE 2:**
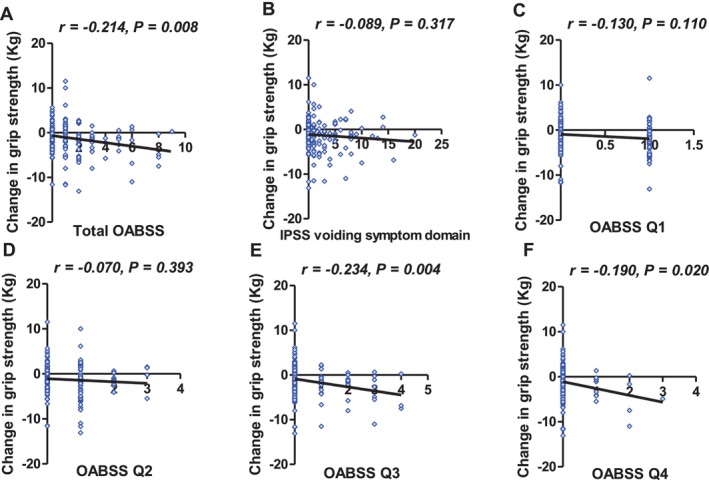
Correlation analyses between lower urinary tract symptoms (LUTS) and grip strength change. (A) Total Overactive Bladder Symptom Score (OABSS), (B) International Prostate Symptom Score (IPSS) voiding symptom domain, (C) OABSS Q1 (daytime urinary frequency), (D) OABSS Q2 (nocturia), (E) OABSS Q3 (urinary urgency), (F) OABSS Q4 (urinary urge incontinence).

**FIGURE 3:**
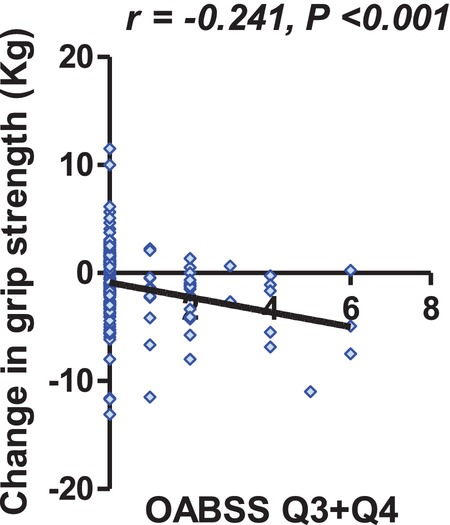
Correlation analyses between OABSS Q3 + Q4 and grip strength change.

The correlation coefficients for the grip strength change and LUTS severity in 2015 are shown in Figure 2. There was a significant negative correlation between the total OABSS and the grip strength change, whereas there was not between the grip strength change and IPSS voiding symptom domain (Figure 2A,B). On the subgroup analyses of the OABSS, we found no significant correlation between the grip strength change and the OABSS Q1 and Q2 (Figure 2C,D). There was a significant correlation between the grip strength change and OABSS Q3 (*r* = −0.234, *p* = 0.004, Figure 2E) and Q4 (*r* = −0.190, *p* = 0.020, Figure 2F). Figure 3 illustrates the relationship between baseline OABSS Q3 + Q4 score and grip strength change over the 4‐year follow‐up period (*r* = −0.241, *p* < 0.001). In contrast, the IPSS storage subscore showed a weak but non‐significant correlation with grip strength change (*r* = −0.151, 95% CI −0.303 to 0.009, *p* = 0.0648).

### 3.3 Multiple Linear Regression Analyses for Grip Strength in 2015 and the Grip Strength Change

A multiple linear regression analysis for the grip strength change showed that the total score of OABSS Q3 and Q4 was significantly correlated (standardized *β* = −0.920, *p* = 0.012, Table 2). When OABSS Q3 and Q4 were entered separately into the multivariable model, Q3 was independently associated with grip strength decline (*β* = −0.93, 95% CI −1.83 to −0.02, *p* = 0.045), whereas Q4 was not (*β* = −0.08, 95% CI −0.85 to 0.69, *p* = 0.84).

**TABLE 2:** Multiple linear regression analysis for the grip strength change.

Variable	Grip strength change	
	Standardized b	*p*
Age	−0.55 (−1.34 to 0.24)	0.170
AMS scale	0.53 (−0.13 to 1.19)	0.120
Serum albumin level	0.15 (−0.51 to 0.80)	0.660
HbA1c	−0.01 (−0.65 to 0.64)	0.980
METs daily exercise	−0.10 (−0.69 to 0.50)	0.740
IPSS voiding symptom domain	0.16 (−0.54 to 0.85)	0.660
OABSS Q3 + Q4	−0.92 (−1.62 to −0.21)	0.012
SMI	−0.48 (−1.21 to 0.25)	0.200
Serum testosterone level	0.35 (−0.29 to 0.99)	0.280
Serum IL‐6 level	−0.04 (−0.63 to 0.56)	0.910
Percent fat mass	0.44 (−0.30 to 1.17)	0.240

Abbreviations: AMS, aging males' symptoms; BMI, body mass index; HbA1c, hemoglobin A1c; IL‐6, interleukin‐6; IPSS, International Prostate Symptom Score; METs, metabolic equivalents; OAB, overactive bladder; SMI, skeletal muscle mass.

## 4 Discussion

This study evaluated the longitudinal association between LUTS severity and decline in physical function in men, using the Community Health Promotion Program database. Although the effect sizes were relatively small (*r* ≈ 0.2) and should be interpreted with caution, we found that the change in grip strength over 4 years was significantly correlated with the baseline severity of urinary storage symptoms, especially with the frequency of UU and UUI. This trend was not observed between grip strength change and baseline voiding symptom severity. Furthermore, our multiple linear regression analysis showed that the total frequency of UU and UUI (total score of OABSS Q3 and Q4) has an independent negative association with the grip strength change. Our results imply that men with more severe storage symptoms tend to experience a more rapid decline in grip strength, regardless of age and other factors.

LUTS is thought to be closely associated with sarcopenia. This relationship is explained by age‐related decline, because these factors are worsened with age [27]. The aging process reduces the number of muscle fibers and motor units, resulting in a decrease in muscle strength both in skeletal and smooth muscle. In the present study, storage symptoms related to OAB, particularly urinary urgency, were associated with subsequent decline in grip strength, whereas voiding symptoms assessed by the IPSS voiding subscore were not. This finding suggests that urgency‐related OAB symptoms may have a more specific relationship with physical function decline than voiding symptoms, which are more closely related to anatomical obstruction such as BPH. Our additional analyses showed that baseline grip strength was not significantly correlated with subsequent changes in IPSS or OABSS over the 4‐year follow‐up period (IPSS change: *r* = 0.065, *p* = 0.435; OABSS change: *r* = −0.027, *p* = 0.747). These findings indicate that baseline muscle strength itself does not predict subsequent changes in LUTS severity. Instead, the observed association appears to be directional, whereby baseline storage symptoms—particularly OAB symptoms—are associated with future decline in muscle strength rather than the reverse.

Chronic low‐grade inflammation has been proposed as a common biological pathway underlying both decline in physical function and LUTS, making it a plausible mechanism linking OAB symptoms to muscle strength decline. Inflammatory processes promote muscle protein degradation and inhibit protein synthesis, thereby contributing to sarcopenia [28]. Previous studies have reported associations between elevated inflammatory markers, such as CRP, tumor necrosis factor‐α (TNF‐α), and IL‐6, and both physical dysfunction and LUTS/OAB [15, 29, 30]. However, our data did not support systemic IL‐6 as the primary mediator in this cohort. In this study, serum IL‐6 levels were neither significantly associated with the 4‐year change in grip strength nor with OABSS severity. The median IL‐6 level in our participants (0.99 pg/mL) was considerably lower than levels reported in studies that found significant associations with LUTS (e.g., 2.80–3.30 pg/mL) [31], suggesting our cohort had a lower overall inflammatory burden. Taken together, these findings indicate that explaining the association between OAB symptoms and muscle strength decline primarily through systemic low‐grade inflammation may be difficult in this relatively healthy cohort.

Several limitations of this study should be acknowledged. First, the baseline grip strength of the participants was relatively high (median 41.4 kg), indicating that the study population cannot be considered frail or sarcopenic. This characteristic is likely attributable to the nature of a voluntary community‐based health survey that tends to attract individuals with high health awareness and relatively healthy lifestyle behaviors. Accordingly, the present study was not designed to investigate frailty or advanced sarcopenia, and interpretations related to these conditions should be made with caution. In addition, baseline LUTS severity was generally mild in this community‐dwelling cohort; the applicability of our findings to clinically symptomatic OAB populations may be limited. Nevertheless, this characteristic also represents an important aspect of the present study. The finding that OAB symptoms were associated with subsequent decline in grip strength even in this relatively healthy and robust cohort suggests that urgency and urge urinary incontinence may reflect early functional vulnerability before frailty or sarcopenia becomes clinically apparent. Second, in the present study, gait function was not significantly associated with LUTS severity (data not shown). This may be because the study population consisted of relatively young and healthy community‐dwelling men, in whom gait function was largely preserved at baseline. Therefore, it remains unclear whether LUTS reflects global or advanced decline in physical function involving multiple functional domains, such as gait performance. Second, the exact reason for the relationship between LUTS severity and grip strength change is not known. The lack of data on low‐grade inflammatory markers other than serum IL‐6 levels, such as hs CRP and TNF‐α, prevents the demonstration of an association between LUTS severity, decline in physical function, and inflammatory markers. Furthermore, the exclusion of 107 participants without a physical examination, who were older than those included in the study, may have introduced selection bias, as these individuals might have had less physical function. Caution is necessary when generalizing the results to other populations due to potential regional bias. Moreover, the study participants may have primarily consisted of individuals with milder comorbidities who were health‐conscious, given their voluntary participation in the health survey. The absolute decline in grip strength over the 4‐year follow‐up period was relatively small (−1.1 kg) and may partly reflect measurement variability. However, this magnitude of decline is consistent with age‐related changes in grip strength reported in population‐based studies, suggesting that the observed decline is within a physiologically plausible range.

In conclusion, this longitudinal community‐based study showed that the severity of urinary storage symptoms, particularly urinary urgency and urge urinary incontinence, independently predicted subsequent decline in grip strength in men.

## Author Contributions

Conceptualization: Teppei Okamoto. Data collection: Teppei Okamoto, Tomoko Hamaya, Hirotake Kodama, Takuma Narita, Jotaro Mikami, Naoki Fujita, Hayato Yamamoto, Atsushi Imai. Formal analysis and investigation: Teppei Okamoto. Writing – original draft preparation: Hiroyuki Sato, Teppei Okamoto. Editing: Tomoko Hamaya, Hirotake Kodama, Takuma Narita, Jotaro Mikami, Naoki Fujita, Hayato Yamamoto, Atsushi Imai. Supervision: Koichi Murashita, Shigeyuki Nakaji, Shingo Hatakeyama.

## Funding

This work was supported by the JST (JPMJCE1302, JPMJCA2201, JPMJPF2210).

## Ethics Statement

The project adheres to the ethical principles outlined in the Declaration of Helsinki, and the utilization of data from the project was granted approval by the Ethics Committee of Hirosaki University School of Medicine (authorization number: 2018‐062). This analysis was also approved by the institutional ethical committee (authorization number: 2024‐093‐1).

## Consent

Participants willingly provided written informed consent and responded to questionnaires regarding their lifestyle and personal information. In addition, an opt‐out approach was implemented, whereby information about the study was publicly disclosed and participants were given the opportunity to decline participation.

## Conflicts of Interest

The authors declare no conflicts of interest.

## Supporting information


**Table S1:** Baseline characteristics between excluded and included participants.

## Data Availability

The datasets generated and analyzed during the current study are not publicly available due to ethical restrictions and privacy protection of participants.
