# Supplementary material for: Overactive Bladder Symptoms as a Predictor of Longitudinal Decline in Grip Strength in Community‐Dwelling Men: A 4‐Year Longitudinal Study
Source: Low Urin Tract Symptoms. 2026 Mar 8;18(2):e70056. doi: 10.1111/luts.70056 (PMC12968119; doi:10.1111/luts.70056)
Supplement: Supplementary file 1 — Table S1: Baseline characteristics between excluded and included participants. [file LUTS-18-e70056-s001.pptx]

## Slide 1
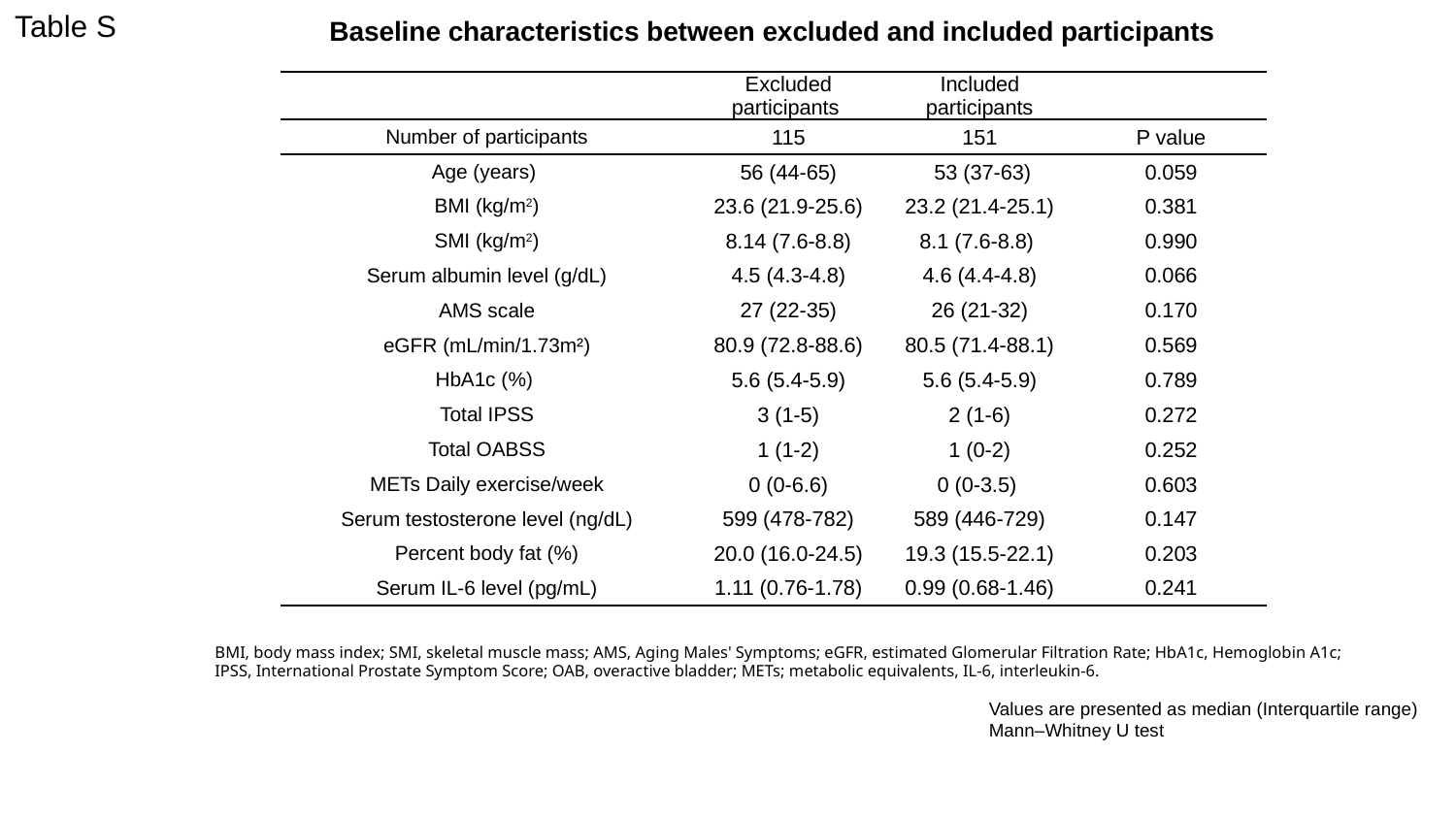

Table S
Baseline characteristics between excluded and included participants
| | Excluded participants | Included participants | |
| --- | --- | --- | --- |
| Number of participants | 115 | 151 | P value |
| Age (years) | 56 (44-65) | 53 (37-63) | 0.059 |
| BMI (kg/m2) | 23.6 (21.9-25.6) | 23.2 (21.4-25.1) | 0.381 |
| SMI (kg/m2) | 8.14 (7.6-8.8) | 8.1 (7.6-8.8) | 0.990 |
| Serum albumin level (g/dL) | 4.5 (4.3-4.8) | 4.6 (4.4-4.8) | 0.066 |
| AMS scale | 27 (22-35) | 26 (21-32) | 0.170 |
| eGFR (mL/min/1.73m²) | 80.9 (72.8-88.6) | 80.5 (71.4-88.1) | 0.569 |
| HbA1c (%) | 5.6 (5.4-5.9) | 5.6 (5.4-5.9) | 0.789 |
| Total IPSS | 3 (1-5) | 2 (1-6) | 0.272 |
| Total OABSS | 1 (1-2) | 1 (0-2) | 0.252 |
| METs Daily exercise/week | 0 (0-6.6) | 0 (0-3.5) | 0.603 |
| Serum testosterone level (ng/dL) | 599 (478-782) | 589 (446-729) | 0.147 |
| Percent body fat (%) | 20.0 (16.0-24.5) | 19.3 (15.5-22.1) | 0.203 |
| Serum IL-6 level (pg/mL) | 1.11 (0.76-1.78) | 0.99 (0.68-1.46) | 0.241 |
BMI, body mass index; SMI, skeletal muscle mass; AMS, Aging Males' Symptoms; eGFR, estimated Glomerular Filtration Rate; HbA1c, Hemoglobin A1c; IPSS, International Prostate Symptom Score; OAB, overactive bladder; METs; metabolic equivalents, IL-6, interleukin-6.
Values are presented as median (Interquartile range)
Mann–Whitney U test
